# Circulating extracellular vesicles in sera of chronic patients as a method for determining active parasitism in Chagas disease

**DOI:** 10.1371/journal.pntd.0012356

**Published:** 2024-11-20

**Authors:** Noelia Lozano, Alexa Prescilla-Ledezma, Eva Calabuig, Maria Trelis, José Miguel Sahuquillo Arce, José Luis López Hontangas, Luis Miguel de Pablos, Mercedes Gomez-Samblas, Antonio Osuna

**Affiliations:** 1 Area of Parasitology, Department of Pharmacy and Pharmaceutical Technology and Parasitology, University of Valencia, Burjassot, Valencia, Spain; 2 Department of Clinical Microbiology and Parasitology, Hospital Universitario y Politécnico La Fe, Martorell, Valencia, Spain; 3 Department of Human Microbiology, Faculty of Medicine, University of Panama, Panama; 4 Department of Parasitology, Biochemical and Molecular Parasitology Group CTS-183, and Institute of Biotechnology, University of Granada., Granada, Spain; 5 Infectious Diseases Unit, Internal Medicine Department, Hospital Universitario y Politécnico La Fe, Martorell, Valencia, Spain; 6 University of Valencia-Health Research Institute La Fe-IIS, Valencia, Spain; 7 Area of Parasitology, Department of Pharmacy and Pharmaceutical Technology and Parasitology, University of Valencia, Burjassot, Valencia, Spain; 8 Joint Research Unit on Endocrinology, Nutrition and Clinical Dietetics, University of Valencia-Health Research Institute La Fe-IIS, Valencia, Spain; The Ohio State University, UNITED STATES OF AMERICA

## Abstract

**Background:**

Chagas disease, once restricted mainly to the Americas, Chagas disease has become a global health problem due to migration from endemic to non-endemic areas. In non-endemic regions, transmission is limited to vertical transmission from infected mothers to newborns or through blood and organ donations. A major challenge in the management of the disease lies in the diagnosis of chronic cases, as blood-borne parasites are often absent and antibodies persist for life, complicating the evaluation of treatment.

**Methodology and main findings:**

This study investigates whether detection of circulating extracellular vesicles (EVs) or their immunocomplexes with host IgGs in the serum of chronic patients with Chagas disease could serve as diagnostic tools and biomarkers of the active presence of the parasite. This method may prove valuable in cases where parasitaemia and other diagnostic tests are inconclusive, especially for assessing treatment efficacy and confirming mother-to-child transmission. Together with exovesicle purification by ultracentrifugation, which is the ‘gold standard’, an affordable and simplified method for the isolation of EVs or immunocomplexes was tested for use in less well-equipped diagnostic laboratories.

EV detection was performed by enzyme-linked immunosorbent assay (ELISA) targeting Trypanosoma cruzi antigens. Positive results were demonstrated in Bolivian patients in Spain, covering asymptomatic and symptomatic cases (cardiac, gastrointestinal or both). The study also examined infected mothers and their newborns. These findings were further confirmed in Panamanian patients with inconclusive diagnostic results.

Moreover, host IgG isotypes that formed immunocomplexes with parasite exovsicles were identified, with IgG2 and IgG4 being predominant.

**Conclusions:**

Our results confirm the usefulness of circulating EVs and their immunocomplexes as markers of metabolically active T. cruzi in chronic infections without detectable parasitaemia, as well as their efficacy in confirming vertical transmission and in cases of inconclusive diagnostic tests.

## Author summary

Chagas disease (CD), or American trypanosomiasis, is considered by the WHO as one of the most important neglected diseases of the American continent, considered as strictly American, there are currently cases of the disease spread all over the world as a consequence of human movements even in areas where the biological cycle of the protozoan parasite responsible for the disease does not occur.

Laboratory diagnostic methods vary between direct tests that denote active parasitemia in blood in the acute phase of the disease and indirect molecular methods applied when the circulating parasitemia disappears in the chronic phase of the disease.

Despite the existence of numerous diagnostic, molecular or immunological tests, the latter being generally easier to perform in diagnostic laboratories, the presence of the parasite always remains in doubt in various situations. Particularly in those patients after treatment since the presence of antibodies to the parasite remain at high titres for years, or in the case of newborn diagnosis in which the mother’s IgG makes specific immunological tests impossible for months.

Therefore, given the need to find biomarkers in chronically ill patients or in mothers and newborns that indicate the active presence of parasites, we have used the purification of parasite extracellular vesicles (EVs) in circulating blood or the immunocomplexes they form with host IgGs, by means of two techniques, the gold standard technique by ultracentrifugation and an adapted technique, easier to apply in diagnostic laboratories, to purify these EVs in order to study the recognition of these EVs by antibodies against the parasite or against specific antigens of *Trypanosoma cruzi* specific antigens.

We believe that this technique can be used when it is necessary to evaluate the efficacy of treatments or in neonatal diagnosis.

## Introduction

Chagas disease (CD), or American Trypanosomiasis, is considered by the World Health Organization (WHO) to be one of the most important neglected diseases in the Americas [1,2], where about 8 million people are affected, with about 15,000 deaths per year due to this disease [3,4].

Until a few decades ago, the disease was considered a strictly American disease, endemic in 21 countries. From southern Texas and New Mexico to Argentina and Chile, today, due to human migratory flows, it is considered to have a worldwide distribution [5]. Cases have been diagnosed in geographical areas where epidemiological conditions do not allow the natural maintenance of the disease, such as North America (central and northern USA and Canada); Europe (countries where the migratory flow from Latin America has been important, especially in Spain, Italy, Sweden), and Australia. Considering that in Spain alone, the number of people affected within the Ibero-American population is estimated at around 55,000 cases [5,6].

CD is caused by the protozoan *Trypanosoma cruzi*, a flagellated protozoan which, in addition to humans, affects domestic and wild mammals, some of which act as reservoirs, and CD should therefore be considered a zoonosis. Hematophagous insects belonging to the family Triatominae (Reduviid) are responsible for the transmission of CD, acting as invertebrate hosts or vectors in the natural transmission of the disease. The flagellate, once ingested with the blood of the infected mammal, multiplies in the midgut of the vector in the form of epimastigotes, which eventually reach the end of the intestine and the rectal ampulla giving rise to metacyclic trypomastigotes or mammal-infective forms.

The development of the disease has a series of distinct stages, an incubation phase, lasting one week to 20 days depending on the vectorial or transfussional route of infection. The incubation phase is usually asymptomatic and lacks specific pathognomonic symptoms. The acute phase occurs in the first weeks after infection with a high blood parasitaemia characterising this acute phase. The acute phase disappears and with it the circulating parasitaemia, approximately three to eight weeks after infection, leading to the chronic phase (Chronic Chagas disease, CCD) which will last for decades with mild to no symptoms and especially with a very low level of parasitaemia. It is estimated that only 30% of cases develop pathognomonic symptoms of the disease [7–9]. The symptoms include cardiac and/or gastrointestinal disorders [10]. These chronic patients (CCD) constitute the greatest epidemiological risk of disease transmission in countries where infection by insect vectors does not occur.

Laboratory diagnostic methods vary from direct tests that denote the active presence of the parasite, such as microscopic observation of the parasite in blood, the microhaematocrit buffy coat [11,12] to xenodiagnosis using triatomines fed on patients and applicable to both the acute and chronic phases of the disease, sometimes including the use of PCR to detect the active presence of *T*. *cruzi* in the gut of the insects used for diagnosis [13], methods rarely or not at all used in in non-endemic countries, or more commonly the use of indirect techniques as different immunological techniques, from rapid immunocomatography tests, indirect immunofluorescence (IIF), enzyme-linked immunosorbent assays (ELISA) with different antigens, Western Blotting (WB), or more recent procedures such as chemiluminescent techniques in which different antigens (native or recombinant) or chimeric antigens are used [14,15].

The variability in diagnostic accuracy has been related not only to the type of technique or antigen used, but also to geographical differences in infected patients, differences in infecting parasite strains or the origin of the diagnostic antigen, or genetic differences between human populations, which may contribute to discrepancies in the sensitivity and specificity of different serological tests [16]. These controversies have led to a series of recommendations by the Pan American Health Organisation (PAHO) and national guidelines [17–19] recommending the confirmatory use of two serological tests in parallel, with a sensitivity of at least 98% [20] or a correct diagnosis of the disease. On the other hand, in treatment efficacy studies or in the case of neonatal diagnosis, it is necessary to demonstrate the active presence of the parasite either by tests that allow visualisation, isolation and growth of the parasites or by other unequivocal tests that show the active presence of parasites in the patient’s biological fluids such as blood, serum or plasma.

The presence of immunocomplexes in patients with CD has been described by some authors both in chagasic patients and in experimentally infected animals [21], attributing some of the pathological manifestations of the chronic phase of the disease to such immunocomplexes [22,23]. Some publications have considered these immunocomplexes as useful tools for diagnosis. For instance, Ohyama et al. (2016) [24] and Petray et al. (1992) [25] studied the parasite antigens present in the serum immunocomplexes of patients affected by CCD using proteomic analysis. In their results, the presence of proteins such as Transialidases or GP63, proteins typical of trypanosomatids (*T*. *cruzi*, *Leishmania* ssp. or *Trypanosoma brucei*), was found [26–28]. Díaz-Lozano et al. (2017) [29] described how these immunocomplexes can be formed by extracellular vesicles (EVs) from the parasite and immunoglobulins from the host, these immunoglobulin-linked EVs were present in the serum of chronic chagasic patients regardless of the pathology of the patients and how these immunocomplexes could be prognostic markers of disease pathology, acting as carriers for a series of parasite-specific proteins, and without orthologues in other Trypanosomatids, such as proteins belonging to the mucin-associated surface proteins (MASP) family. MASP is a multigene family of approximately 1,300 proteins [30–32] which have a high variability, except in two regions of identical nucleotide sequences in all the proteins of the multigene family, the region corresponding to the C-terminal 5’ sequence (C-term) that is coding for a signal peptide (SP), and the N-terminal 3’ region. The rest of the sequence is hypervariable which makes the MASP proteins different from each other [33].

EVs are small membrane-coated vesicles released into the extracellular environment by all types of cells, both eukaryotic and prokaryotic, and are classified according to their size, biogenesis and composition, including exosomes (~30–100 nm), ectosomes (~100–500 nm) and apoptotic vesicles (> 500 nm) [34]. EVs act as carriers including a wide variety of lipids, proteins, different populations of RNAs, ssDNA, and/or metabolites. EVs participate in cell-cell communication processes in an endocrine, paracrine or juxtacrine manner [35]. They can participate in numerous cellular functions from immunomodulation, antigenic presentation [36], modify cellular niches or be carriers of genetic markers and gene transfer between cells [37] and can be useful in molecular diagnostic systems [38].

The production of EVs by *T*. *cruzi* was first described by Da Silveira et al. (1979) [39] and the role of these EVs in promoting parasitism has been demonstrated, [40–42] both at organ and cellular level, due to their ability to induce changes in the cells which they interact [43] modulating cell physiology such as the cytoskeleton of cells, modifying cytosolic calcium levels, altering the permeability of cells, modifying the cell cycle or the transcriptome [44,45]. Proteomic studies of EVs released by trypomastigote forms show a series of parasite-specific proteins such as MASP proteins, or transialidases, which together with cruzipain constitute specific antigens capable of being recognised by the immune system of affected patients [29,46–49].

As EVs constitute specific carriers of both proteins and nucleic acids, Lozano et al. (2023) [48] recently determined how EVs from the plasma of chronic CD patients could be used in the molecular diagnosis of Chagas disease, demonstrating how these EVs carry nucleic acids from the parasites and can be amplified using either strictly mitochondrial KDNA-specific probes or nuclear DNA probes capable of amplifying parasite satellite DNA. This fact demonstrates that in these chronic patients, with little or no circulating parasitemia by traditional techniques, there must be metabolically active forms of the parasite, such as trypomastigote or amastigote forms, capable of releasing EVs, we have evaluated the possibility of using EVs derived from the parasite or forming circulating immunocomplexes in the serum of patients with CCD from two different geographic regions of America, as new diagnostic biomarkers that denote the active presence of *Trypanosoma cruzi*.

The aim was: i) to determine whether the detection of circulating EVs present in the sera of chronic patients with Chagas disease, typed and clinically classified according to symptomatology, contain circulating immunocomplexes formed by parasite EVs and IgGs of the host, denoting the biochemically active presence of the parasites; ii) we also attempted to characterize the different isotypes of IgGs that form these immunocomplexes, which may help in the future to purify these immunocomplexes by immunochromatographic techniques; iii) we tried to develop an easy and affordable technique for the isolation of immunocomplexes, which avoids having to use exovesicle purification by ultracentrifugation (the gold standard) and which can be applicable in diagnostic laboratories where specialized instrumentation is lacking; iv) we tried to develop a technique to be used in those cases where it is required to demonstrate the presence or absence of metabolically active parasites, as in the case of analysis of the efficacy of treatments or newborns where traditional techniques for the study of parasitemia encounter difficulties due to the low number of parasites in blood or other biological fluids.

The detection of EVs was performed by immunological techniques using either an immunoserum against *Typanosoma cruzi* or antibodies against highly parasite-specific proteins such as the signal peptide of the MASP proteins, a region of constant sequence in this large family of proteins.

## Materials and methods

### Ethics statement

For the study of Bolivian patients residing in Spain, we have the permissions of the following Ethics Committees: Ethics Committees of HUyP-La Fe with the numbers HUyP-La Fe, Valencia, Spain (2016/0866), and of the University of Granada, Human University of the Granada Ethical Committee, Spain, with number n°: 672/CEIH/2018.

All participating patients received an ‘informed consent’ document to be signed, which is included in the supplementary material. The document includes a section for the “clinical study in children”, where the notification to the Public Prosecutor’s Office is included, as required by Spanish legislation for the participation of minors, and which must be signed by the parents or legal guardians of the minors.

For the study of the patients from Panama, permission was obtained from the Ethics Committee of the University of Panama, Faculty of Medicine with number 2015-310V1 and from the Human Ethics Committee of the University of Granada with number 672/CEIH/2018. All Panamanian patient participants received an informed consent document which they were required to sign in accordance with Panamanian regulations.

### Study populations

In this study, two populations of patients with CCD from Latin America were examined.

The first population comprised 92 patients of Bolivian origin who currently reside in Spain. These individuals live in the Spanish city of Valencia, where they were diagnosed and underwent medical follow-up at the Hospital Universitario y Politécnico La Fe (HUyP-La Fe) Valencia, Spain. The Bolivian samples included 92 patients who underwent screening tests, applying three serological assays: i) LiaisonXL murex (Diasorin), ii) rapid test (SD Bioline Chagas Ab Rapid, Abbott 49FK10), and iii) IFA kit (Trinity Biotech). All these tests, as mentioned earlier, are routinely applied for the diagnosis of individuals suspected of being affected by CCD at the HUyP-La Fe. Of the 92 Bolivian patients, 63 were adults with CCD, comprising 49 (77.8%) females and 14 (22.2%) males. Additionally, there were 16 newborns from mothers with CD and 13 CD-negative individuals, selected as controls.

The 63 patients with positive immunological tests or PCR (Lozano et al 2023) were summoned to the Infectious Diseases Unit, Internal Medicine Department, for control and monitoring of CD. A physical examination, an electrocardiogram, a chest x-ray, an echocardiogram, a barium enema and esophagogram were performed and it was registered whether they presented any cardiac or digestive symptoms at the hospital electronic medical chart. Patients with possible cardiac involvement also underwent cardiac magnetic resonance imaging and 24-hour Holter monitoring. Of these of the adult patients, 24 had indeterminate symptoms, including 16 pregnant women. There were 20 patients with cardiac involvement, 14 with gastrointestinal pathology, and 5 with both cardiac and gastrointestinal symptoms. In the case of the 16 newborns, samples were analyzed by PCR at birth, repeated at one month and at nine months of age.

The second group, and as a proof of concept of the results obtained with patients diagnosed in Spain, consisted of 106 individualized sera of CCD patients, coming from Panama a country considered endemic for CD since 1930. The samples had been used in a previous work by our research group [50] and in which the DTU of the parasites that produced the infection had been typed. Sampling was conducted in both rural areas, where patients have continuous contact with vector insects, and urban areas within Panama City. Informed consent procedures, surveys, and blood sample collection were carried out for all patients who voluntarily chose to participate in the study. Of the studied Panamanian population, a total of 106 potential CD patients were analyzed, with 78 out of the 106 (73.58%) residing in the rural community of Chararé, located in the mountainous region of the Las Margaritas, Chepo district, Panama province (coordinates 9.243640, -79.059162). Screening was conducted using three serological tests: i) rapid test (SD Bioline Chagas Ab Rapid, Abbott 49FK10); ii) WB using the methodology previously described by Saldaña et al. (1995) [51]; iii) ELISA Chagatest (Wiener lab 1293257) [50]. Only individuals who tested positive in two out of these three tests were considered positive. Out of the initial 106 individuals, 53 were identified as positive (33 from rural areas and 20 from urban areas) and were used to validate the presented diagnostic methods. A selection of 25 sera randomly selected, from the individuals previously diagnosed as positive in Panama [50] by screening with at least two of the serological tests described above and clinically evaluated in the hospitals of Chepo and Santo Tomas, after an electrocardiographic examination and thoracic radiography and clinically classified as Chagas positive. In all cases, negative parasitization by *Trypanosoma rangeli* and *Leishmania ssp*. was evaluated, which in the case of Panamanian leishmaniasis is of cutaneous pathology. In order to calculate the cut-off value of the absorbances obtained from the circulating Evs in the sera of the patients. Fifty sera were used as shown in S3 Table, from individuals negative by immunological and PCR tests for CD, from the same geographical region. Of these sera, five showed syphilis pathology and two showed leprosy pathology. None of these cases showed infection by Leishmania ssp. or *T*. *rangeli*.

For the validation of immunocomplexes detection after dissociation of the immunoglobulins present in the sera of CCD patients, a total of 117 sera from both patient populations were used. This included 92 sera from the population diagnosed at HUyP-La Fe, and a selection of 25 sera from the 53 individuals previously diagnosed as positive in Panama through screening with the three serological tests described above. This selection included two sera negative for ELISA Chagatest, two negative for WB, two negative for the rapid test, one negative for both ELISA Chagatest and WB, 16 positives for all three screening tests, plus a negative reference serum and an existing positive reference serum in our laboratory’s serum bank.

### Immunological tests used for Chagas disease patient diagnosis

As previously mentioned, the immunodiagnostic tests employed for diagnosing patient populations varied based on their availability at the hospitals where the patients were recruited and analyzed.

For patients of Bolivian origin diagnosed at HUyP-La Fe, analysis was conducted using the LiaisonXL murex kit (Diasorin). This kit employs a chemiluminescence immunoassay with recombinant antigenic proteins (multi-antigen). The methodology followed was in accordance with the manufacturer’s recommendations. As a second diagnostic test, an immunofluorescence assay (IFA) kit (Trinity Biotech) was used, following the manufacturer’s guidelines.

For the diagnosis and confirmation of the Panamanian patients, the initial test used was the rapid test (SD Bioline Chagas Ab Rapid, Abbott 49FK10). To conduct the assay, 100 μL of serum were deposited into the sample well along with 50 μL of assay buffer. Test interpretation was performed visually after a 15-minute incubation period at room temperature.

Another diagnostic test used for CD diagnosis in this population was a commercial ELISA test for antibodies against *T*. *cruzi*, specifically the ELISA Chagatest (Wiener lab 1293257). The assay was carried out and validated following the manufacturer’s instructions; accordingly, serum samples were diluted to a concentration of 1:20.

All serum samples from Central American patients underwent an antigen recognition test for the parasite by patient immunoglobulins using WB, following the methodology described by Saldaña et al. (1995) [51] and subsequently published by Ledezma et al. (2020) [50]. For diagnostic use, the transferred strips were incubated with patient serum (1:100) for 2 hours. Subsequent treatment after the washes was done with peroxidase-conjugated rabbit secondary antibodies (Dako, anti-Human IgA, IgG, IgM, Kappa, Lambda/HRP, ref: P0212) diluted 1:700 in PBS, for 1 hour, as previously described [33].

### Isolation of immunocomplexes by ultracentrifugation “Gold Standard” and dissociation of purified immunocomplexes (Ig-EVs)

The purification of circulating immunocomplexes (Ig-EVs) in the serum of patients was carried out following methods previously described by Díaz-Lozano et al. (2017) [29], and Lozano et al. (2023) [52] through a mixed procedure of filtration through 0.45 μm filters followed by differential ultracentrifugation at 110.000 xg for 2h in in microcentrifuge tubes (Hitachi No 1508) at 110,000 × g for 2 hours at 4 °C in a CP100NX centrifuge (Hitachi Koki, Tokyo, Japan) with a fixed-angle rotor P70A.

After this centrifugation stage, the pellets containing the immunocomplexes were washed three times by ultracentrifugation in sterile filtered PBS and evaluated through nanoparticle tracking analysis (NTA) and transmission electron microscopy, as described in Lozano et al. (2023) [52] and Cornet-Gomez et al 2023 [53].

In order to separate the EVs present in the sera from the immunoglobulins forming the immunocomplexes, the pellet containing the immunocomplexes was resuspended in 90 μl of PBS containing a cocktail of protease inhibitors without EDTA (Roche, ref: 11836170001). Subsequently, to the suspension containing the immunocomplexes, 650 μl of 0.1 M glycine-HCl at pH 4 were added and incubated for 15 minutes at room temperature. This suspension in glycine-HCl pH 4 buffer was ultracentrifuged again at 100,000 x g for 1 hour to separate circulating EVs in the pellet and the immunoglobulin solution that forms the immunocomplexes in the supernatant. The supernatant was aliquoted and pH-neutralized with Tris-HCl Buffer, pH 10, Antigen Retriever (Sigma T6455) containing 0.1% Glycerol, frozen, and kept at -20 °C until use for IgGs purification by affinity chromatography using Protein G and subsequent determination of the isotype, as described later. The pellet obtained from ultracentrifugation was resuspended in PBS, centrifuged again at 100,000 x g, the supernatant removed, and resuspended in 80 μl of 0.1 M bicarbonate buffer (pH 9.6) containing protease inhibitors without EDTA (Roche, ref: 11836170001).

### Isolation and concentration of EVs through filtration with centrifugal concentrators

As an alternative method, for the concentration of EVs and immunocomplexes present in serum in order to avoid using the ultracentrigugation techniques described above, which may be unaffordable for hospital and health center laboratories, the method using protein concentrators previously described by Orrego et al. (2021) [54] and Ramírez et al. (2018) [55] was followed, with some modifications.

Briefly, patient serum (1 ml) was diluted with 5 ml of ultrafiltered PBS. Subsequently, centrifugation at 1,500 x g for 10 minutes was conducted, and the resulting supernatant was filtered through a pore size of 0.45 μm. Following this initial filtration and centrifugation, the filtered supernatant underwent a further centrifugation step at 3,500 × g for 20 minutes. The final supernatant, diluted with PBS was applied to Vivaspin protein concentrator (Sartorius Lab Instrument, Goettingen, Germany) with a separation cut-off size of 100K (100,000 MWCO), which were centrifuged at 6,000 × g for 1 hour at 4 °C. The retained volume in each concentrator was collected, aliquoted, and stored at -80 °C until use.

### Determination of the size of EVs and purified immunocomplexes

The hydrodynamic size distribution of the purified immunocomplexes obtained by either method described above was measured by NTA (Nanoparticle Tracking Analysis), using an instrument equipped with a sample chamber, a 405-nm laser, and a high-sensitivity complementary metal-oxide-semiconductor (CMOS) camera. The samples were diluted in 0.22 μm filtered PBS up to 1 ml and then loaded into the chamber. Three 60 s videos, in Brownian mode, were recorded and analyzed for each sample with NTA 2.3 image-analysis software (NanoSight Ltd., Amesbury, UK). The mean size distribution was calculated as a mean of three independent size distributions. This methodology follows the procedures previously described by Retana-Moreira et al. (2021) [48] and by Lozano et al. (2023) [52] and by Cornet-Gomez et al. (2023) [53].

### Use of animals for the Production of antisera and authorization by the animal welfare and ethics committee

The use of animals for obtaining antisera was carried out in accordance with the guidelines set forth in the Spanish Government Regulation (Royal Decree RD1201/05) and the European Union Directive (European Directive 2010/63/EU). It was approved by the Ethics Committee of the University of Granada and by the Regional Government authorities of Andalusia (Junta de Andalucía) with the number ES1802100000038 in 2017.

### Preparation of polyclonal antibodies against *T*. *cruzi*

Three four-week-old male Wistar rats were intraperitoneally immunized with 20 μg of a total extract from *T*. *cruzi* Pan4 trypomastigotes per dose, combined with Freund’s adjuvant, to produce polyclonal anti-*T*. *cruzi* antibodies. The parasite extract was derived from 10^9^ trypomastigotes obtained from cell cultures, which were previously washed and concentrated by centrifugation, following the procedure described by Cornet et al. (2023) [53].

Antibody titers in serum samples were determined on a weekly basis after the first two immunizations using an indirect ELISA. At the end of the immunization period (8 weeks), the animals were euthanized in an isoflurane atmosphere. Whole blood samples were obtained by cardiac puncture.

To design the synthetic peptide corresponding to the consensus sequence of the signal peptide (SP) of MASP proteins, the methodology described by Díaz-Lozano et al. (2017) [29] was followed. A consensus sequence (MAMMMTGRVLLVCALCVLWSVAADG) (S3 Fig) was used, which was synthesized by LifeTein (USA, LLC) with four branches joined by lysine residues.

The production of polyclonal antibodies against the synthetic peptide corresponding to the signal peptide sequence of MASP proteins was carried out in three four-week-old male Wistar rats with 100 μg of the MASP SP peptide per dose, respectively. Before the first immunization step, a blood extraction was performed in all cases to obtain preimmune control serum. Antibody titers of anti-MASP SP and anti-*T*. *cruzi* extract sera were verified by an indirect ELISA in multiple well microtiter plates (Nunc, Thermo Fisher) coated with 5 μg of the antigen/well in 0.1 M bicarbonate coating buffer (pH 9.6). Sera with titers greater than 1:6,400 were selected and stored at -80 °C, diluted 1:1 with glycerol (Molecular Biology grade, Sigma) until use, and were referred to as anti-MASP SP antisera or anti-*T*. *cruzi* total antisera.

### Electrophoretic confirmation of *Trypanosoma cruzi* antigens present in circulating EVs in the plasma of patients

In order to recognize the *T*. *cruzi* antigens present in the EVs isolated from the plasma of patients affected by Chagas disease, the western blot methodology described in Retana Moreira et al [26] was followed, for which the purified EV proteins were precipitated in acetone at -20 °C overnight. They were centrifuged at 13,000 x g for 10 min at 4 °C and washed twice with cold acetone. After the acetone residue was removed, the precipitated proteins were quantified using the Micro BCA Protein Assay Kit (Thermo Scientific, ref: 23235). 30 μg of these proteins were loaded into the wells of 12% SDS-PAGE gels after electrophoresis and subsequent transfer to PVDF membranes (BioRad, Hercules, CA, USA). The membranes were immersed in blocking buffer (PBST plus 4% skimmed milk). Incubated at 4°C for 12h with a 1:1000 dilution of polyclonal anti-*T*. *cruzi* antibody, the membranes were washed and incubated with a secondary antibody anti rat (1:10000) peroxidase-conjugated goat antibody (Sigma-Aldrich, ref: A9037) for 1h at room temperature. Detected bands were visualized using Clarity ECL Western Substrate (BioRad, Hercules, CA, USA) on a ChemiDoc Imaging system (BioRad, Hercules, CA, USA).

### Antigenic recognition by ELISA by anti-MASP SP or anti-*T*. *cruzi* immunosera from EVs isolated from patient sera

For ELISA assays, Nunc 96 multi-well plates (Thermo Fisher Scientific) with a volume of 100 μl per well were coated with a concentration of 5 μg/μl of proteins from a lysate of EVs, in RIPA buffer, isolated from each serum sample in 100mM carbonate/bicarbonate buffer (pH 9.6). The protein concentration was determined using the Micro BCA Protein Assay Kit (Thermo Scientific, ref: 23235), following the instructions. Plates were incubated under shaking for 8 hours at 4 °C. After adsorption, the plates were twice washed with PBST to remove unbound antigens.

Subsequently, 250 μl of a freshly prepared blocking solution were added and incubated at 4 °C under shaking overnight. The plates were washed again with PBST. Then, 100 μl of rat *T*. *cruzi* primary serum (1:2000) or anti-MASP SP (sera with titters higher than 1:6400) diluted 1:500 in PBS were added to each well, and the plates were incubated for 2 hours under shaking at room temperature. After antibody interaction, the plates were washed at least three times in PBST, and 100 μl of peroxidase conjugated polyclonal goat anti-rat IgG (Sigma-Aldrich, ref: A9037) at a dilution of 1:1000 in PBS were added, followed by incubation at room temperature under shaking for 1 hour.

Following incubation with the secondary antibodies, the plates were washed four times, and O-phenyl-diaminobenzidine plus 30% H_2_O_2_ (1 μl/ml) (Sigma-Aldrich) was added to 0.05 M phosphate-citrate buffer, pH 5.0, as a peroxidase substrate. The plates were further incubated for 15 minutes at 27 °C. The reaction was halted with a solution of 0.1 M 2 N H_2_SO_4_, and absorbance was measured at 492 nm using an ELISA Multiskan Spectrum reader (Thermo Fisher Scientific).

To determine the cut-off value of the EVs, these were individually purified from the plasma of Chagas disease-negative 13 control sera of the same origin and geographic region that were challenged by ELISA with anti-MASP SP and anti-*T*. *cruzi* immunosera, as described in the previous paragraph. And where, three negative sera from Spain, two positive for *Leishmania infantun* and one positive for leprosy, diseases in which cross-reactions with CD have been described, were included.

The cut-off value was calculated as the average OD at 492 nm plus three times the standard deviation value of the samples (mean of the OD + 3 x SD).

### Isotyping of immunoglobulin G (IgG) forming immunocomplexes with EVs isolated from CD-Positive plasma

IgGs were separated from the immunocomplexes by treatment with 0.1 M glycine-HCl at pH 4 and subsequent ultracentrifugation as described above. The IgGs were purified by chromatography using Protein G HP SpinTrap/Ab Spin Trap columns (GE Healthcare Life Sciences, 28-9031-34), following the manufacturer’s instructions. Briefly, after removing the storage buffer from the columns, the column was equilibrated with 600 μL of binding buffer composed of 20 mM sodium phosphate (Sigma-Aldrich, 255793) in PBS. Washed twice with 600 μL of binding buffer to remove those sample components not bound to protein G from the column, the columns were eluted by centrifugation for 2 min at 100xg in 40 0ul of elution buffer composed of (0.1 M Glycine-HCl, pH 3.0). The columns were eluted in 2 ml vials containing 15 μL of basic pH buffer (Tris-HCl buffer, pH 10, Antigen Retriever). Elution as in the previous steps was performed by centrifugation at 100 × g for 2 minutes, repeated twice, resulting in a final eluate volume of 800 μL for each of the immunocomplex samples from each of the sera.

Once the purified IgGs were obtained, they were quantified using the Micro BCA Protein Assay Kit (Thermo Scientific, ref: 23235).

Isotyping of different IgG isotypes, as well as immunoglobulin subclasses, was performed via ELISA following the previously described methodology. Primary antibodies included IgG immunoglobulins (1:1,000) from rat (anti-human IgG2a ThermoFisher), mouse (anti-human IgG1 and anti-human IgG2 of Sigma, anti-human IgG2b of Biomedicals, and anti-human IgG3 and anti-human IgG4-HRP of Abcam) were incubated for 1 hour at room temperature under shaking. As a secondary antibody labeled with HRP peroxidase (except for IgG4, which is already labeled with HRP), a 1:1,000 dilution of anti-rat (Sigma-Aldrich, ref: A9037) or anti-mouse (Dako, ref: P0447) was added and incubated for 1 hour at room temperature with gentle stirring.

Washes were performed as described above with PBST, and 100 μl of peroxidase as substrate and incubated for 20 min at room temperature under shaking in the dark. Finally, 50 μl of 3M HCl stop solution in distilled water were added, and the reading was carried out at 492 nm in a MultiskanSpectrum spectrophotometer (Thermo Fisher Scientific).

### Statistical analysis

The Shapiro test was used for testing normality of the distribution of the data. Normal distributed data are expressed as mean (± standard deviation) and were compared with the ANOVA and Tukey test. Nonparametric data are expressed as median (interquartile range) and were compared with the Mann-Whitney U test and the Kruskal-Wallis test. Nonparametric related data was studied with the Wilcoxon signed-rank test and the Bonferroni-corrected significance level method was applied.

Statistical analyses were performed with Rstudio and a p < 0.05 was considered significant.

## Results

### Sample selection. CCD patients of Bolivian origin living in Spain

Of the patients of Bolivian origin diagnosed at HUyP-La Fe, all were positive in the three immunological techniques mentioned (LiaisonXL murex (Diasorin), rapid test (SD Bioline Chagas Ab Rapid, Abbott 49FK10), IFA kit (Trinity Biotech); hence they were used in the subsequent studies (S1 Table). Of these, 63 were randomly selected from various situations and pathologies, including indeterminate symptoms, cardiac pathology, gastrointestinal disturbances, and both cardiac and gastrointestinal disturbances. Additionally, 16 pregnant women with CD and their newborns were included (summary in S2 Table).

### Ultracentrifugation vs Filtration for the detection of *T*. *cruzi* Antigens in EVs from Sera of CCD patients

The protein concentrator method used was designed, as already indicated, to facilitate the purification and concentration of EVs from serum or plasma in order to facilitate instrumentally and technologically the collection of EVs from serum. We compared the use of protein concentrators to purify EVs from plasma with the gold standard method for the purification of EVs (ultracentrifugation).

The results obtained from the characterization of the NTA purified EVs with both methods are shown in S1A and S1B Fig and in Table 1.

**Table 1:** Results of the determination of the size and amount of EV protein obtained by the two purification procedures. (*) (p-value <0.00001).

Method	Size mean (nm)	Mode	D90	Protein concentration (ug/ul) (*)
**Ultracentrifugation**	209.8	166.5	290.2	27.9 ± 10.8
**Filtration**	240.4	208.4	378.1	9.5 ± 8

With the method in which protein concentrators were used, as shown in the Table 1, larger peaks were obtained, possibly aggregates of the EVs with each other (S1A and S1B Fig). The total protein concentration was statistically higher when the ultracentrifugation method was used for purification (27.9 ± 10.8 μg/μl) compared to the alternative filtration method (9.5 ± 8 μg/μl) (p-value <0.00001) (S2 Fig).

In order to test the antigenic recognition in EVs purified by protein concentrators and the gold standard of EV purification by the two immunisera (anti MAM Sp and anti Tc (total)), 24 individual serum samples were randomly selected from a total of 63 clinically characterized positive samples for CD from patients residing in Spain.

Among these 24 samples individually selected samples, there were 7 pregnant women. Of the 24 samples, (S1 Table), 14 patients had indeterminate symptoms, while 5 had gastrointestinal symptoms and 5 had cardiac pathology. Each sample was treated by both ultracentrifugation-ultrafiltration and concentrator purification to separate circulating vesicles, as described in Materials and Methods.

**Fig 1:**
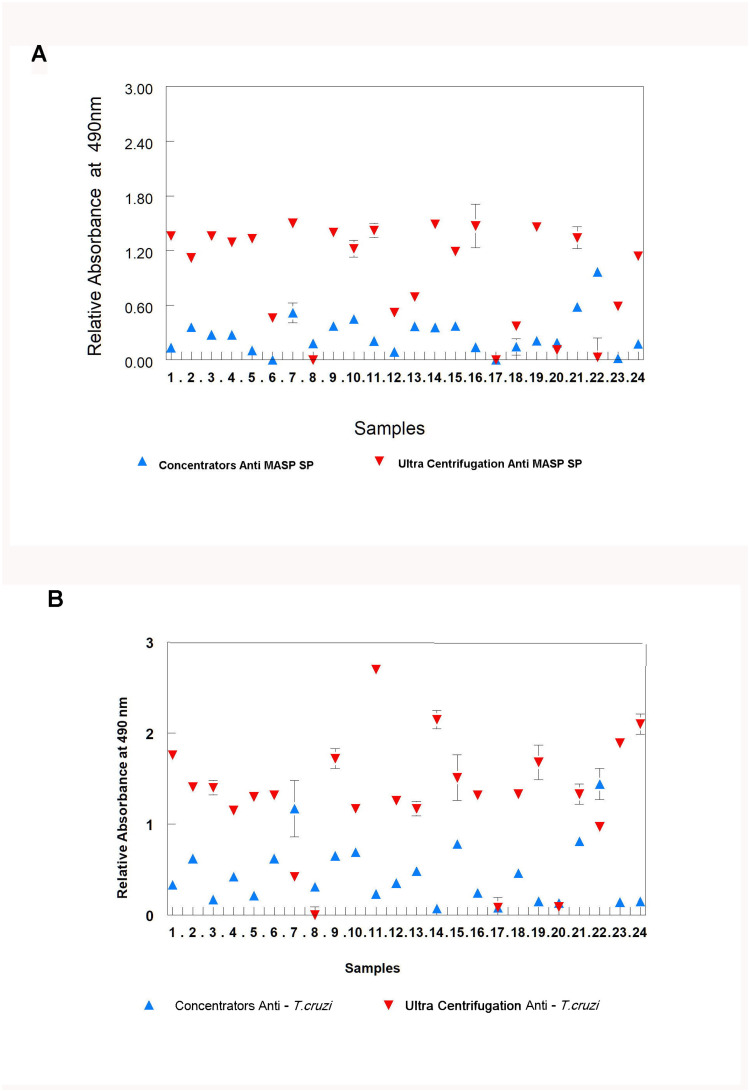
Antigenic recognition of EVs obtained from sera of patients with CCD by concentrators vs differential centrifugation. Relative absorbance was calculated as the absorbance values at 490 nm, minus the mean absorbance value of the cut-off value, obtained by the same immunoassays and treatment against EV from individuals without CD. A. Developed with anti-MASP SP immunoserum. B. Developed with anti-*T*. *cruzi* immunoserum.

Purification of EVs by ultracentrifugation and detection with anti-MASP SP detected those 21 patients had relative absorbance values above the cut-off threshold, while for the remaining three, the absorbance was close to 0. This same pattern was replicated when the EVs were purified by the filtration method (Fig 1A).

Employing the anti-*T*. *cruzi* for EV detection, 23 patients showed absorbance values above the cut-off threshold when EVs were purified through both ultracentrifugation and filtration (Fig 1B).

For the same EV isolation method, no significant differences were found between the two immunosera used. The Wilcoxon rank sum test yielded W = 208.5 and a p-value >0.05 for the ultracentrifugation method, and W = 374 with a p-value >0.05 for the filtration method.

For both markers, absorbance values were significantly higher when applying the ultracentrifugation technique to purify EVs from serum samples of CCD patients compared to purification via the filtration technique. Specifically, anti-*T*. *cruzi* (W = 94, p-value <0.05) and anti-MASP SP (W = 99, p-value <0.05) showed ELISA elevated absorbance levels. These findings were derived using the Wilcoxon rank sum test with continuity correction, as the variables did not adhere to a normal distribution. This suggests a consistent trend favoring the ultracentrifugation method over filtration in both markers (Fig 1A and 1B).

### Determination of antigenic recognition in EVs from Sera of CCD patients resident in Spain by Immunosera, anti-*T*. *cruz*i and anti-MASP SP

The patients were categorized into groups based on their symptoms: indeterminate symptoms (24 total patients), cardiac pathology (20 total patients), gastrointestinal symptoms (14 total patients), and those presenting with both cardiac and gastrointestinal pathologies (5 total patients).

**Fig 2:**
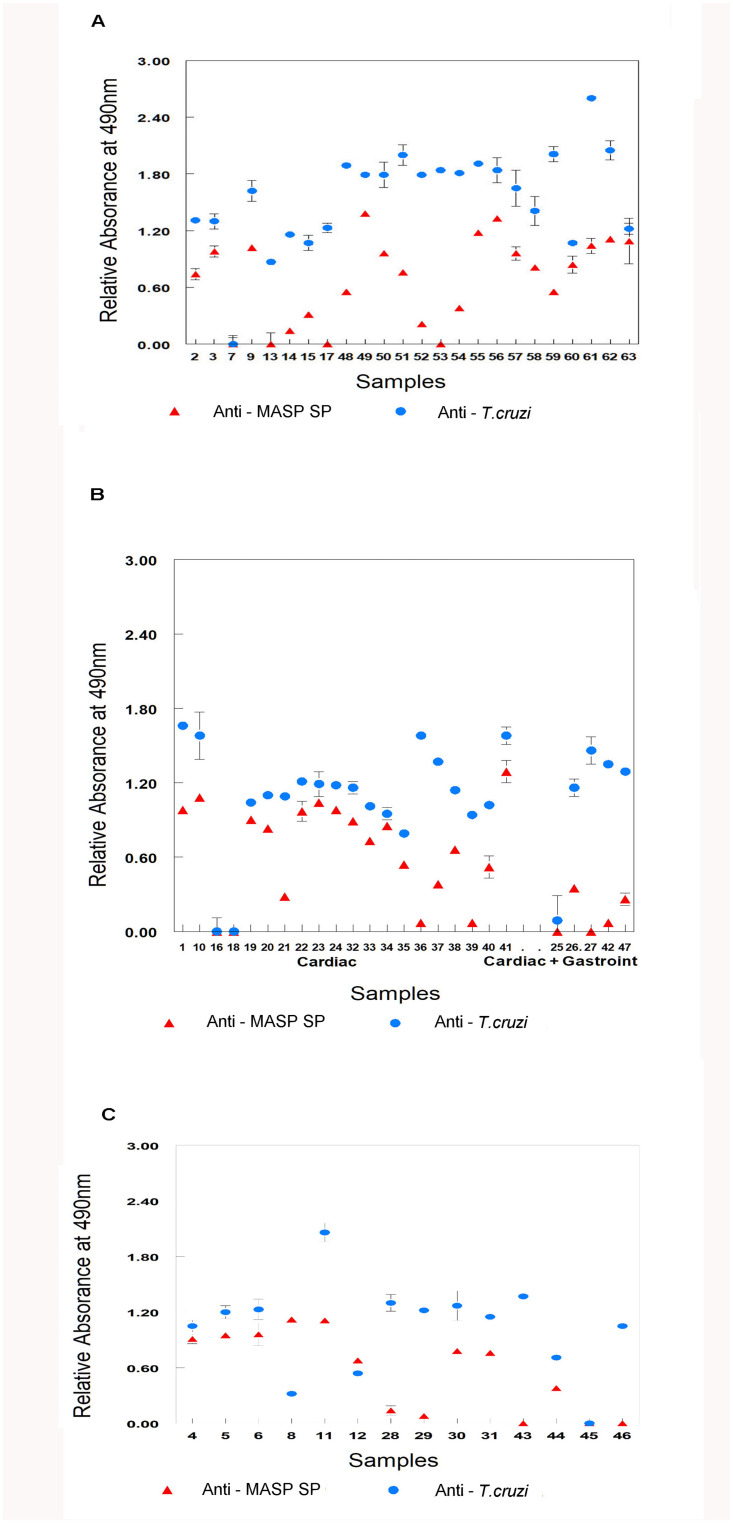
Antigenic recognition by anti-*T*. *cruzi* and anti-MASP SP immunosera of EVs obtained by ultracentrifugation from sera of Bolivian patients with CD diagnosed in Spain and classified by symtoms. The absorbance value at 490 nm is represented on the y-axis and samples from different patients are represented on the x-axis. The absorbance results are the net values after subtracting the cut-off value from the total absorbance A. Patients with CD and indetermined symthoms. B. Patients with CD and cardiac disorder, and with cardiac and gastrointestinal symptoms together. C. Patients with gastrointestinal symptoms.

Of the 24 samples from indeterminate patients, EVs reacted in 23 (95.8%) samples against anti-*T*. *cruzi*. The absorbance levels were under the cut-off value in a 42-year-old man (number 7), who had a previous positive PCR in 2010 and was treated, reactivity of EVs obtained to both immunosera gave absorbance values below the cut-off value (Fig 2A and Table 2).

**Table 2:** A summary of the results obtained with anti-*T*. *cruzi* and anti-MASP SP in the different clinical situations.

Patients	Total number	anti-*T*. *cruzi*			anti-MASP SP		
		% Positivity	Mean	Mediana	% Positivity	Mean	Mediana
**Indeterminates**	24	95.8	1.83	1.9	83	0.83	0.93
**Cardiac**	20	90	1.21	1.16	90	0.72	0.82
**Gastrointestinal**	14	92	1.19	1.28	78	0.66	0.79
**Cardiac and Gastrointestinal**	5	80	1.31	1.38	60	0.21	0.13

When anti-MASP SP immunoserum was used against EVs, 20 of the 24 indeterminate patients samples (83%) had absorbances higher than the cut-off value. The absorbance levels were lower than the cut-off value in four patients, number 7 and 13 had positive PCRs and was treated, number 17 and 53 had a negative PCR and number 53 was a pregnant woman (Fig 2A and Table 2).

Of the 39 symptomatic CD patients, 35 (89.7%) samples were positive with anti-*T*. *cruzi*, and 32 (82,1%) samples with anti-MASP SP (Table 2).

Two cardiac patients (patients numbers 16 and 18), 1 gastrointestinal (patient number 45) and 1 cardiac and gastrointestinal patient (patient number 25) presented lower absorbance levels with anti-*T*. *cruzi* than the cut-off values (Fig 2B and 2C).

Two cardiac patients (patients number 16 and 18), 3 gastrointestinal patients (patients number 43, 45 and 46) and 2 cardiac and gastrointestinal patients (patients number 25 and 27) had lower absorbance levels with anti-MASP SP than the cut-off values (Fig 2B and 2C).

The results of antigen recognition in all patient groups, (cardiac, gastrointestinal and cardiac plus gastrointestinal pathologies) shows that the detection of EVs was more effective with anti-*T*. *cruzi* immunoserum than with anti-MASP SP (Mann-Whitney U 993.5, p < 0.00001) for all the patients with different disorders.

When we evaluated the antigenic recognition obtained in EVs using anti-*T*. *cruzi*, significant differences were observed in the absorbances obtained depending on the pathology shown by the patients (Kruskal-Wallis test, chi-square = 14.536, df = 3, p-value = 0.00226). The group of patients with indeterminate symptoms showed significantly higher absorbance values compared to the absorbance obtained with circulating EVs from the cardiac and gastrointestinal patient groups (Bonferroni-corrected significance level method, p-value <0.05). However, this trend was not statistically significant when the anti-MASP SP immunoserum was used (Kruskal-Wallis test, chi-square = 6.1322, df = 3, p-value = 0.1054).

### Sample selection. Patients in Endemic areas, Panama

The rapid tests applied allowed us to obtain quick and reliable results, capable of having a first sweep of the population under study, mainly in the rural area where health resources are scarce, since their results are qualitative or semiquantitative and the samples do not require any type of equipment, testing system or specialized refrigeration. In the rural patients of the Chararé community, 87.8% (29/33) were positive, while 75% (15/20) of the urban patients analyzed presented positive results in the rapid test (S1 Table).

**Fig 3:**
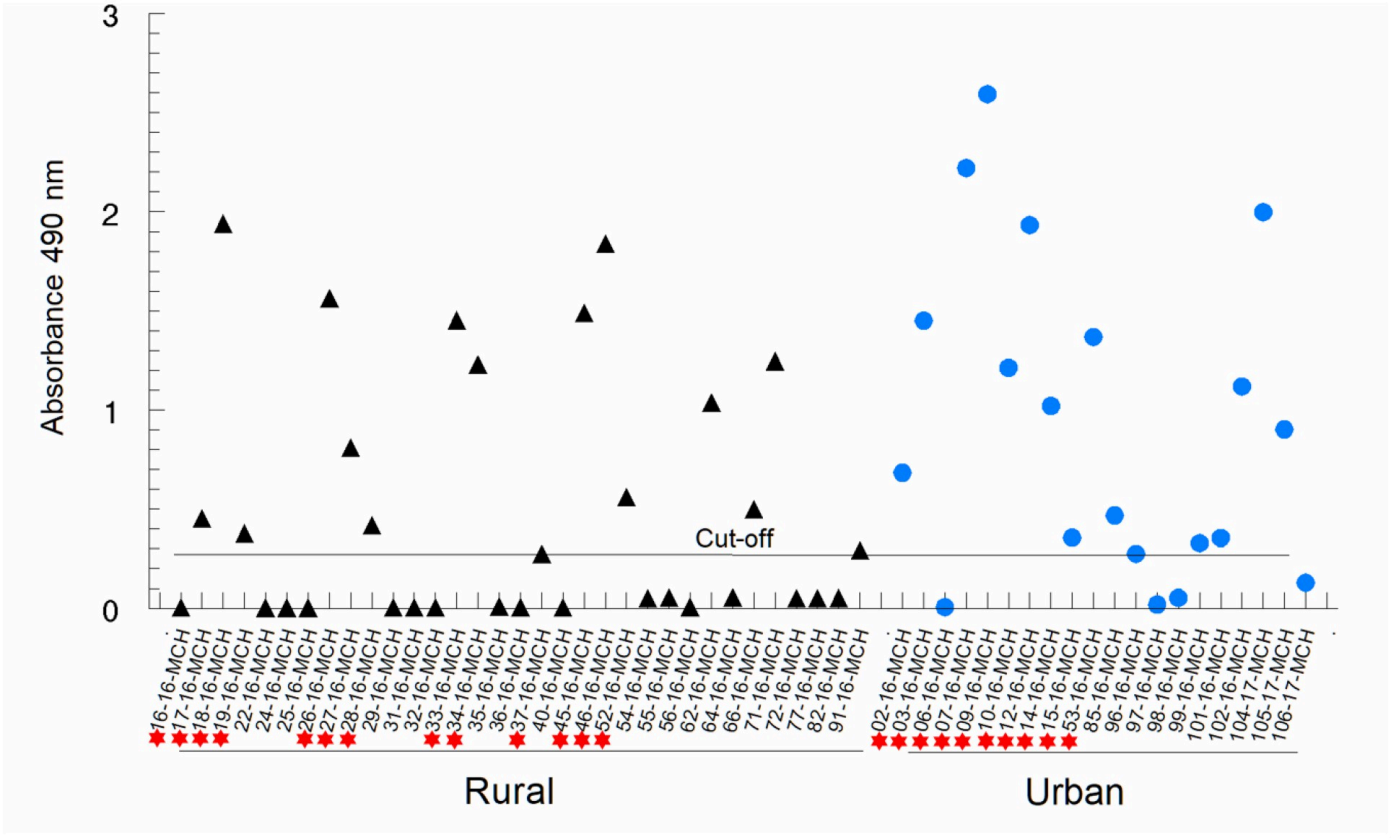
Absorbance obtained by ELISA with the different serum samples. **Comparison between rural and urban samples of patients diagnosed with CD in the Panamanian population.** Black triangles for patients living in rural areas and blue circles for urban patients in Panama City. Red asterisks show sera that were selected for proof-of-concept for Fig 4 used in subsequent experiments with antigen recognition in circulating EVs. The horizontal line represents the cut-off value of the negative sera obtained from the mean of the absorbances of these sera plus three times the standard deviation of the means of these negative sera (mean absorbance of negative sera +3 x SD).

A second commercial ELISA test (Wiener lab) was conducted in 53 chronic Chagas patients. Among them, 54% (29/53) tested positive, while 6% (3/53) were classified as clearly inconclusive due to their absorbance values falling within the "grey" zone or at the cut-off value (cut-off = 0.3). Additionally, 40% (21/53) tested negative. Serum samples from a rural population displayed a 42% (14/33) positivity rate for the aforementioned ELISA test method, while sera from urban patients selected from the hospital showed a 75% (15/20) positivity rate. All serum samples were evaluated in triplicate, and the results were recorded (Fig 3 and Table 3).

**Table 3:** A summary of the results obtained with commercial tests and our proof of concept test.

Test %	Positive	Negative	Inconclusive
**Rapid test**	83	17	0
**Wiener ELISA**	54	40	6
**WB**	90	0	10
**anti-T. cruzi ELISA**	100	0	0

The WB analysis reveals distinct antigenic bands in positive sera (25, 30, 45, 52, 70 kDa), while the remaining bands are regarded as nonspecific for CD diagnosis. S4A Fig illustrates results from patients in urban areas, where 90% (18/20) tested positive, and 10% (2/20) yielded inconclusive results. In contrast, patients from the rural community of Chararé (S4B Fig), exhibited an 84% (28/33) positivity rate, with 15% (5/33) showing indeterminate results (Table 3).

### Detection of circulating parasite EVs from the sera of CCD patients from an endemic country (Panama) by anti-*T*. *cruzi* immunoserum. proof of concept

**Fig 4:**
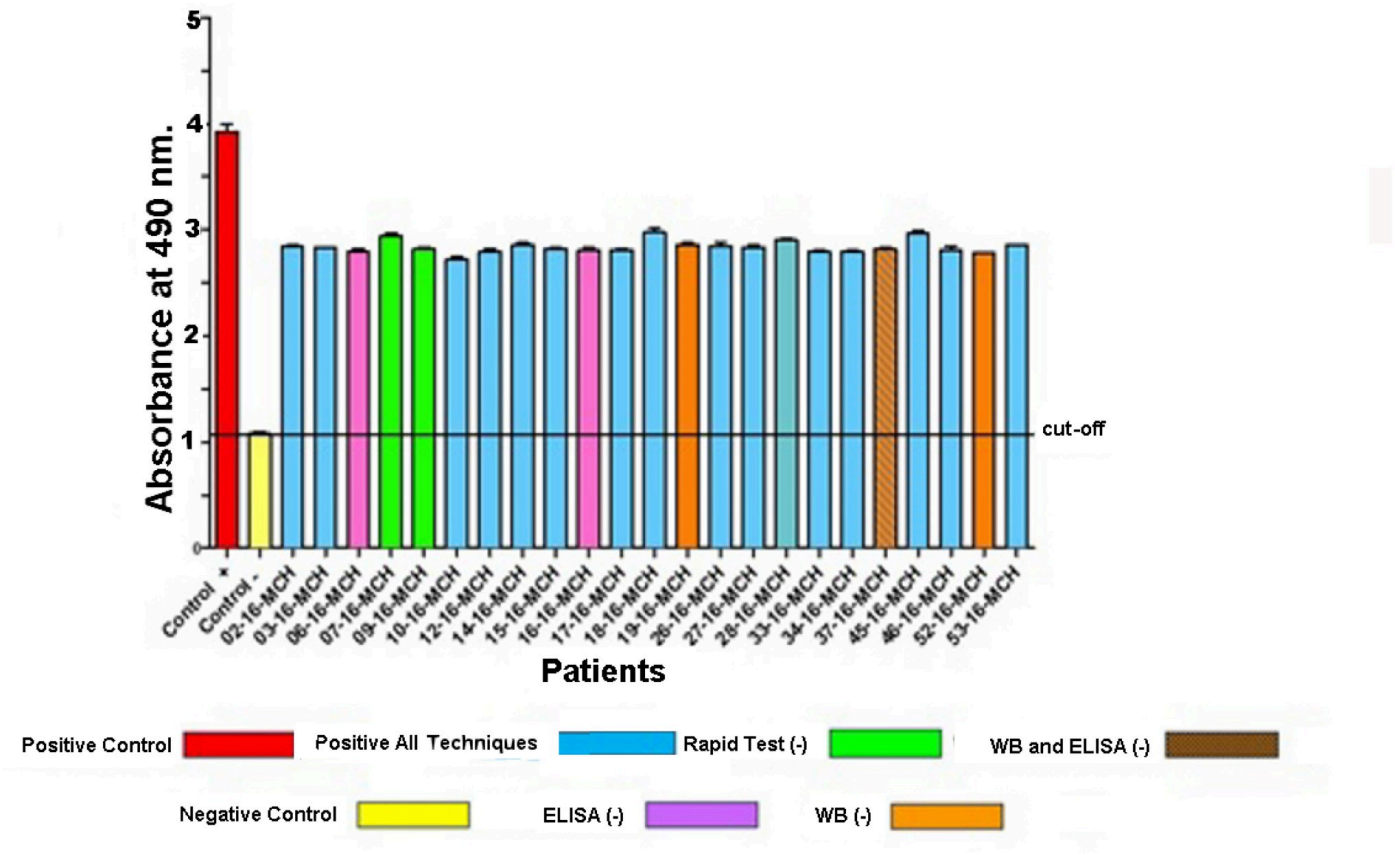
Detection by ELISA of EVs with *T*. *cruzi* antigens in the serum of patients whose diagnosis by conventional diagnostic systems was inconclusive. Red: positive control; yellow: negative control; blue: patients with CCD positive by PCR; Green: patients with CD but negative by rapid test; Brown: patients with CD and negative by WB and Wiener ELISA test; Purple: patients with CD negative by Wiener ELISA test; Orange: patients with CD positive by rapid test and Wiener ELISA but negative by WB.

As proof of concept to evaluate the utility of using circulating EVs as an indicator of the active presence of the parasite in sera where immunological diagnostic systems had been inconclusive, we decided to purify EVs from a total of 25 (16 positive for all techniques; 2 negative for the rapid test; 2 negative for the Wiener ELISA test; 2 negative for WB and one negative for WB and ELISA) as indicated in the text and caption of Fig 4 including a positive control and a negative control. This includes all diagnostic situations of these patient sera of Central American CCD patients from Panama that were in such a situation despite testing PCR positive. Once the circulating immunocomplexes from the serum were purified and any accompanying IgGs that could form immunocomplexes were eliminated, these EVs were confronted with anti-*T*. *cruzi* immunoserum. Negative controls consisted of EVs extracted from panel of 50 serum samples from immunologically negative individuals who lacked symptomatology after medical evaluation of these individual sera from the same geographical region as described in S3 Table and above, but lacking the disease, and treated with a similar procedure as the sera under study.

Fig 4 shows that anti-*T*. *cruzi* antisera recognize antigens in EVs isolated from the sera of 23 patients from Panama who presented inconclusive or negative in commercial diagnostic tests for CD, including WB results. In all cases, the absorbance obtained was higher than the cut-off value obtained from the pool of sera from non-infected individuals.

### Detection of circulating parasite EVs in sera of pregnant Bolivian Women and their infants

**Fig 5:**
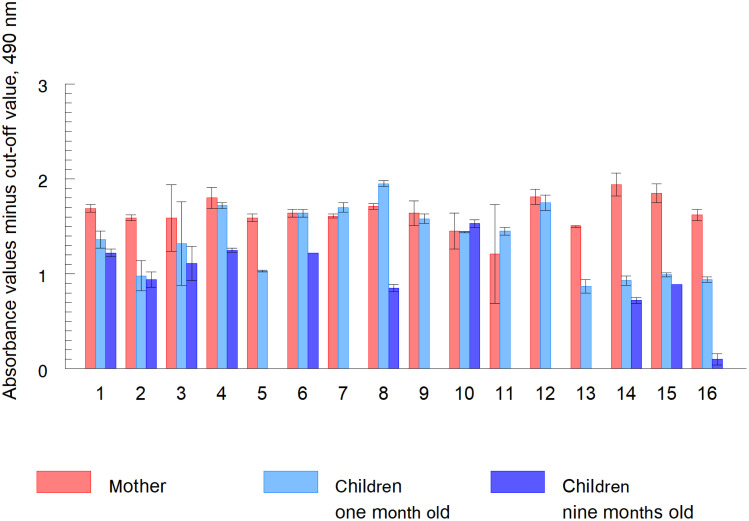
*Trypanosoma cruzi* antigenic recognition in EVs obtained from sera of mothers with CD and their children by anti-*T*. *cruzi* immunoserum. The absorbance value at 490 nm is represented on the y-axis and samples from 16 different mothers and their children are represented on the x-axis. The absorbance results are the net values of subtracting the absorbance of the cut-off value from the absorbance obtained from the samples.

The study on the presence of EVs in pregnant women who tested positive for CD and sequentially tested for reactivity of EVs in their babies at 1 month and subsequently at 9 months after birth revealed that the mean absorbance value at 490 nm for anti-*T*. *cruzi* in the babies at the first month was 2.08 (95% CI: 1.77–2.27), whereas at 9 months, the obtained global result was 1.24 (95% CI, 0.74–1.61). Furthermore, a significant correlation was found with the Spearman test between absorbance values at 1 month and 9 months (Rho 0.72; p-value = 0.009) (Fig 5).

In samples corresponding to children number 5 and 13, the absorbance values were equal to or below the cut-off value using the immunoserum against total *T*. *cruzi* antigen at nine months after birth (Fig 5).

Samples from patients number 7, 9, 11 and 12 at 9 months of age could not be collected as they did not return to the hospital for follow-up of the mother or child.

Sample number 16 corresponds to a child who received treatment two months after birth due to the positive PCR of the mother during pregnancy and subsequently of the child one month after birth. The treatment consisted of Benznidazole, administered orally in three doses for 60 days, 10 mg/kg per day for the child. The mother received the same drug administered orally in two doses for 60 days at 5–7.5 mg/kg per day. At the beginning of the treatment, lactation was interrupted. The absorbance after recognition of the EVs by the anti-*T*. *cruzi* immunosorbent serum at 9 months after birth was very low in this child, treated and with interrupted breastfeeding.

Children in samples 5 and 13 showed significantly lower absorbance levels (similar or lower than the cut-off value at 9 months) between samples taken at 1 and 9 months of age in contrast to samples from children who still showed elevated absorbance at 9 months, (1, 2, 3, 4, 6, 8) although they experienced lower absorbance values compared to those obtained from mothers or at 1 month after birth, except for the child in sample 8, where the absorbance at 1 month was higher than that of the mother. The HSD Tukey test revealed a p-value <0.001 (Fig 5).

In sample 10, the values of circulating antigens in the parasite EVs, were maintained from the first month to the ninth month (HSD Tukey test revealed a p-value >0.05), while in the rest of the samples the absorbance values decreased with respect to those obtained at one month after birth.

The samples from mothers 1, 2, 14, and 16 tested positives in the PCR. While mothers 2, 7, 9, 11, 14, 15 and 16 were treated, the children of these mothers tested negative in the PCR at 2 months after birth. Except for number 16, who tested positive in the PCR and received treatment as indicated above (S2 Table). S1 Table shows the peculiarities of each of the cases in detail.

### Study of IgG subclasses in the immunocomplexes from Chronic CD patients (Bolivia and Panama) by ELISA

After purifying circulating immunocomplexes (IgGs-EVs) from the serum of CD patients using the ultracentrifugation method described above, we isolated the IgG antibodies that are part of the immunocomplexes to characterize the subclasses of IgGs forming them.

**Fig 6:**
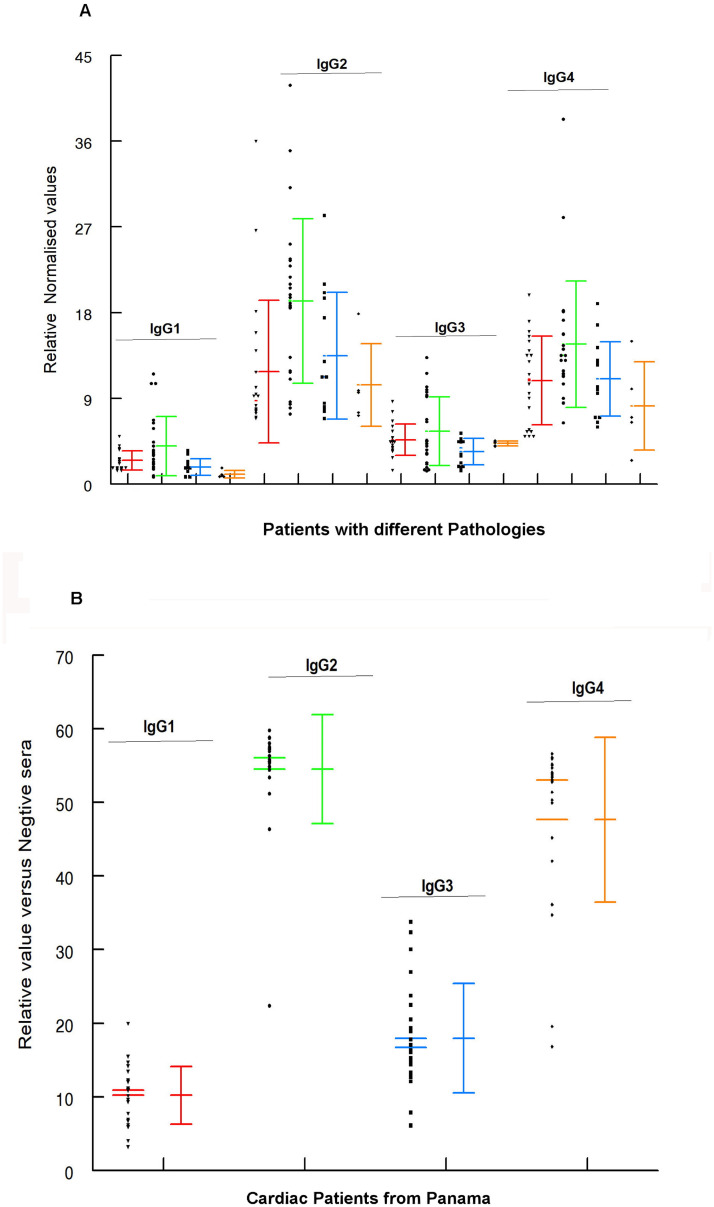
Boxplots illustrating the relative absorbance values measured at 490 nm for each IgG subclass isolated from immunocomplexes obtained from patients with CD. A. Mean values of patients originating from Bolivia with different pathologies. Red: cardiac pathology; Green: indeterminate patients; Blue: gastrointestinal pathology; Orange: combined cardiac plus gastrointestinal symptoms. B. Patients with CD originating from Panama. Mean and SD are represented for each isotype. Red: IgG1; Green: IgG2; Blue: IgG3 and Orange; IgG4.

The results of the Bolivian patients in Spain are represented in Fig 6A. In this analysis, significant differences were observed in the absorbance values between the different IgG subclasses when compared with the different pathologies (ANOVA, Isotypes: p-value <0.0001; Pathology: p-value <0. 0001). Specifically, IgG2 and IgG4 isotypes exhibited statistically higher levels (Tukey HSD test with 95% CI: diff = 12.11, p-value = 0 and diff = 9.36, p-value = 0, respectively), while IgG3 did not show significant differences compared to the other subclasses (Tukey HSD test, p-value >0.05).

When comparing Bolivian patient groups categorized as indeterminate and symptomatic (including cardiac, gastrointestinal, and combined cardiac plus gastrointestinal pathologies) via ANOVA (p-value <0.05), significant differences were found for each isotype. In all cases, the indeterminate patient group exhibited significantly higher levels compared to the diagnosed and pathologically affected patient group, as indicated by the following statistics IgG1 (Tukey test with 95% CI: diff = -1.946763, p-value >0.0001); IgG2 (Tukey test with 95% CI: diff = -6.996506, p-value >0.0001); IgG3 (Tukey test with 95% CI: diff = -1.387083, p-value >0.01) and IgG4 (Tukey test with 95% CI: diff = -4.088365, p-value >0.001).

All the patients from Panama graphed in Fig 6B were diagnosed with cardiac conditions. Interestingly, significant differences were found between all variables (IgG1, IgG2, IgG3, and IgG4) when compared to each other, following adjustment for multiple comparisons using the ANOVA method (p-value <2e-16 ***). The mean values of the IgG2 and IgG4 isotypes were significantly higher than those of IgG1 and IgG3 (Tukey HSD test, p-value >0.05). However, the absorbance values of IgG2 and IgG4 were found to be similar for these patients (Tukey HSD test, p-value >0.05).

## Discussion

The search for specific biomarkers for the diagnosis and prognosis of Chagas disease (CD) continues to be a research challenge to identify the presence of the parasite and the status as well as the prognosis of the development of the disease and that are capable of determining the response to treatment [1,56–60].

While in the acute phase the diagnosis with parasitological techniques confirms *T*. *cruzi* parasitism, the disappearance of the flagellate forms from the bloodstream together with the sustained presence of anti-*T*. *cruzi* antibodies throughout life during CCD constitutes a drawback for a serological evaluation to study the effectiveness of treatments [57], the risk assessment in blood or organ donors or the confirmation of infection in neonates.

In addition, the variability of the results of immunological tests based on the detection of antibodies, where the same patient may present disparity of results depending on the type of test to which he/she is submitted [61], a consequence of possible cross-reactions, the diversity of antigens due to the genetic variability of the parasite [62] and even the geographical differences of the parasite strains used [16,19,63] represents a challenge in CCD diagnosis. All this means that there are national and international recommendations, PHAO or WHO, on the need to perform several immunological assays and that there should be no discrepancies in the positivity of these assays before diagnosing patients as positive [2].

Moreover, standardisation of methods to demonstrate the metabolically active presence of the parasite in affected individuals is necessary in both experimental and clinical evaluation of treatment efficacy. This also applies to the confirmation of parasitism in newborns born to mothers with the disease, and where the earliest possible treatment will allow these children to be cured [58,64] or in cases of follow-up treatment [65,66] as well as in cases of organ and tissue transplant donors who confirm the absence of the parasite in donors from endemic regions, or who have spent significant time in these areas [67,68].

Among the methods that denote the active presence of the parasite in patients, especially designed to detect blood trypomastigote forms in the acute phase of the disease, are the visualization of blood smears stained with Giemsa, or biological enrichment techniques for those cases with low parasitemia; xenodiagnosis with the vector or direct inoculation of blood to laboratory animals, are not applicable in diagnostic laboratories especially in regions where vectorial transmission does not take place. Other techniques based on the isolation and purification of blood trypomatigote forms such as Strout’s technique [69] are easy to develop, but this particular technique requires a high blood volume of about 10 ml, from which, by means of two centrifugations, the blood protozoa can be concentrated and subsequently visualized microscopically. A variant where the volume of the blood sample is significantly reduced to 0.3–0.6 ml and applicable both in the acute phase and in studies of vertical transmission to neonates is the method developed by Freilij 1983 or microhematocrit concentration method [11,64,70]. All these techniques are limited to concentrate and visualize by buffy coat the presence of blood parasites, potentially infecting forms that for handling, are subject in the EU and USA to biosafety standards for handling level 3 in the EU and 2 in the USA [71] which implies avoiding the use of glass or metal and sharp material, which precludes the use of glass microcapillaries to carry them out. On the other hand, these techniques would only detect parasitemia above 50 tryp/ml) and not the presence of parasitism in cells and tissues with quiescent amastigotes and the absence of blood forms, which often cause therapeutic failure [72,73].

In other biological models such as quiescent muscle cells [74] secretion of EVs by such cells could be ascertained, suggesting the possibility of production of EVs released by such amastigotes forms.

A recent review of diagnostic techniques was compiled by Schijman A et al 2024 [75]. Among the most recommended serological methods to confirm Chagas disease are those that use trypomastigote excreted-secreted antigens (TESA) to detect antibodies from the patient, which react with proteins or glycoconjugates released by *T*. *cruzi*, thus consisting of the response to the set of excretion products of the trypomastigote forms of the parasite [76–80].

Although serological assays (ELISA and immunoblot) using TESA are very sensitive, they are still tests that evaluate the titers of antibodies against parasite antigens with the existence of cross-reactivity for patients infected by *Leishmania* ssp [81,82].

Proteomic studies of the TESA antigen reveal the presence of proteins already described in other analyses of the proteome of EVs and particularly Transialidases, GP63, highlighting the presence of retrotransposon hot spot proteins (RHS), that Bautista-Lopez et. (2017) [81] identified and characterised for use as diagnostic markers. In a previous study carried out in our group [48], it was found that the exosomes of trypomastigote forms derived from cell culture showed that 22% of the total protein types corresponded to Transialidases belonging to the groups I-VIII proposed by Freitas et al. (2011) [83] and Nardy et al. (2016) [84], these enzymes being found on the surface of the EVs [49] carrying in turn the cysteine protease Cruzipain and the non-orthologous metalloprotease GP63, likely to be responsible for the cross-reactions observed when TESA is used as a diagnostic antigen, since it is found in both *Leishmania* ssp and *T*. *brucei* [85,86]. Subsequently Nagarkatti et al. (2020) [87] developed an antibody against a sequence of the Tc_517 peptide present in the *T*. *cruzi* secretome whose presence they use as a biomarker in serum for *T*. *cruzi* infection.

The use and usefulness of EVs in the diagnosis of various diseases including infectious diseases such as latent tuberculosis has been published recently [88–90]. Immunocomplex cargoes in patients with CD include Transialidase proteins or GP63, proteins characteristic of trypanosomatids [24]. When these immunomplexes were sequenced, the presence of Transialidase proteins or GP63, proteins characteristic of trypanosomatids, was found. The presence of circulating immunocomplexes consisting of parasite EVs and antibodies against the parasite in the circulating blood of chronic CD patients was described by Díaz-Lozano et al. (2017) [29].

Analysis of circulating immunocomplexes found in the serum of chronically ill patients revealed recognition of the signal peptide (SP) by immunogold techniques under MASP SP electron microscopy in 45.19% of the EVs forming these immunocomplexes, and by immunohistochemistry a maximum absorbance in immunocomplexes from chronically ill patients with digestive pathology [29].

The diagnostic use of PCR and real time qPCR using these immunocomplexes from patients with chronic Chagas disease was recently published by Lozano et al. (2023) [52], demonstrating that these circulating immunocomplexes carry and preserve the DNA from the parasite nucleus as well as from the kinetoplast (KDNA) of mitochondrial origin of the protozoan. EVs are capable of carrying and preserving both DNA and RNA of cellular origin [54,91].

In the present work and as a proof of concept, immunocomplexes with the EVs of the parasite in the serum of chronic Chagas disease patients were isolated. For their isolation and purification, the purification of EVs by ultracentrifugation (with a diameter of 209 nm with a mode of 166 nm) was compared with another methodologically and economically simple method, based on the use of single-use protein separators/concentrators (with a mean particle diameter of 240 nm with a mode of 208 nm). With the latter method, the concentration of total proteins decreased slightly with respect to those purified by ultracentrifugation, perhaps due to the absorption of liquids with dissolved proteins by the matrix of the filtration equipment (S2 Fig). These protein concentrating filters have already been used in the purification of EVs in different biological fluids or culture media [54,92–94].

Our results indicate that the choice of technique depends on our specific objective and the technological facilities of the laboratory to isolate circulating EVs in serum and determine the presence of biological material of the active forms of the parasite.

That is, if our objective is to obtain a higher signal provided by circulating EVs, the method of choice would be ultracentrifugation, since a higher amount of protein is obtained in these EVs.

In order to check the presence of the IgG subclasses that form them in the immunocomplexes purified after dissociating the immunoglobulins as described in Material and Methods, these were characterised by ELISA. The results are shown in Fig 6A and 6B, where it can be seen that, in both the Bolivian and the Panamanian patients, IgG2 and IgG4 were the highest titres of the IgGs forming the immunocomplex. The determination of immunoglobulins in Chagas disease has been studied by several authors. Brodskyn et al. (1989) [95] studied IgGs in Chagas disease, suggesting that the immune clearance of *T*. *cruzi* is due to antibodies located in the IgG isotype, particularly in the IgG2 subclass. Similarly, in an experimental study carried out by Spinella et al. (1989) [83], the main IgG subclass found was IgG2a, reaching 10 times the control level especially in the chronic phase of the disease. This would indicate that some of the EV antigens, possibly glycosylated, would stimulate the antibody response and must be recognised by IgG2.

The lower recognition by the anti-MASP SP could be explained by the data obtained by Díaz-Lozano et al. (2017) [29] where it was observed that only 45.19% of the EVs isolated from the trypomastigote forms presented gold tags when performing immunochemistry under TEM and the number of tags per EV was 1.41 ± 0.65, i.e., only approximately half of these EVs carried the recognised epitopes of the highly specific peptide of *T*. *cruzi* and belonging to what could be considered immature MASP proteins. However, in the proteome of EVs from trypomastigote forms there are 524 proteins, of which 250 are specific for trypomastigote forms [48]. Therefore, the chances of the epitopes of these proteins being recognised is significantly higher than those present in the signal peptide of the MASP proteins.

As proof of concept of the use of circulating EVs in serum forming immunocomplexes, as an indicator of the presence of material from metabolically active parasites, and based on the results obtained with the different diagnostic tests in the Panamanian patients: i) sera positive for all three diagnostic tests; ii) with those where the immunological diagnosis was negative or inconclusive with the different methods tested, including the rapid tests, the Wiener ELISA test (Fig 3) or the WB (S4 Fig), tests with circulating EVs would be recommended to confirm active parasitization.

As indicated above, and in order to test in a series of patients in whom the different traditional diagnostic methods were not conclusive, a total of 23 individualized sera were selected in which positivity had already been proven by PCR performed at the University of Granada in whole blood samples transported with guanidine, in which even DTUs causing the infection were determined [50]. From parallel samples of sera from these patients, circulating exovesicules were extracted by ultracentrifugation. In that panel of sera, there were seven in the above conditions (negative with any of the three techniques or inconclusive). As can be seen in the graph in Fig 4, the absorbances obtained when confronted with EVs to the anti-*T*. *cruzi* immunoserum are shown, all the selected sera gave absorbance values higher than the cut-off value obtained with EVs from the negative control subjects. This demonstrates the presence of metabolically active parasites in all the cases analyzed.

Congenital Chagas disease has now acquired epidemiological relevance, especially after the insect vector control campaigns carried out in many endemic countries [96], and currently remains a crucial challenge for both endemic and especially nonendemic countries, where this form of transmission, along with transfusion or transplantation, would be the only way of spreading the disease in these countries far from vector transmission [97–99]. However, due to the neglected nature of the disease and persistent barriers to access diagnosis, treatment and care, the prevalence in pregnant women and their newborns may be underestimated [10,64,100].

An estimated 1.12 million women of childbearing age are infected by the *T*. *cruzi* parasite [56,64], where the prevalence of vertical transmission approaches 5% [101]. The incidence of congenital Chagas disease is estimated to be between 8,000 and 15,000 cases per year in Latin America [64].

Of the Bolivian patient population assessed in this study, 16 were pregnant women (S2 Table) who tested positive for Chagas disease. Four of them had been diagnosed by PCR prior to pregnancy and seven of them treated with Benzinidazole, (the standard treatment prescribed was Benznidazole, administered orally in three doses for 60 days at 7.5 mg/kg per day for mothers and 10 mg/kg per day orally in children, who tolerated the treatment better). although numbers 14, and 16 did not undergo treatment control as some of them were PCR positive again when they became pregnant, had circulating immunocomplexes isolated from the serum of their infants at one month after birth and at 9 months (S1 Table). It is noteworthy that the umbilical cord blood was not available in any of these cases at the time of birth, as would have been desirable. In the days prior to delivery, some patients (1, 2, 14 and 16) were PCR positive, while only the son of mother 16 was PCR positive and was treated together with his mother, starting two months after birth, for which he was withdrawn from breastfeeding.

The results obtained from PCR in newborns are considered inconclusive due to the limited amount of blood collected, and DNA purification, carried out at the hospital, by automated DNA purification methods [52].

In all cases, the absorbance obtained with the vesicles purified from the sera from both mothers and offspring gave higher values than the cut-off value obtained with EVs from individuals negative for the disease.

Of note is the decrease in absorbance in the infants at one month after birth with respect to that obtained in the mothers, except in cases 7 and 8. and as already indicated, samples were not available from the infant of patient 7, since the mother did not return with him to the nine-month follow-up.

In the case of baby number 10, the absorbance at one month was maintained with respect to that obtained in his mother, increasing the absorbance at 9 months with respect to the values obtained from the other two samples, including the mother’s sample. Although at one month and later he was PCR negative, in a medical check-up one year after birth and before returning to his country of origin, he tested positive in the PCR test and treatment was recommended. Therefore, it could be considered as a false negative for PCR, due to the problems mentioned above, shortage of blood for analysis or shortage of purified DNA.

Sample 16 was different, i.e., both mother and baby tested positive for PCR in the study performed at one month after birth. Both were subjected to treatment two months after birth and breastfeeding was interrupted. In this case, and perhaps as a consequence of the treatment, the values of parasite EVs decreased at 9 months, with the result that the abbsorbances decreased considerably until they were slightly higher than the cut-off value. It should be remembered that the values represented on the ordinate axis are the values of the mean absorbance of the sample, minus the mean value of the cut-off.

This decrease in absorbance could be considered indicative of the efficacy of the treatment, by decreasing the active forms of the parasite and thus the release of circulating *T*. *cruzi* EVs recognized by the anti-*T*. *cruzi* immune serum.

During normal pregnancy, the presence of EVs in the fetal circulatory system and communication between the mother and the growing fetus occur through the exchange of EVs produced by both the mother and the fetus [102,103]. Exosomes derived from the placenta may represent a mechanism by which the placenta communicates to induce maternal adaptations to pregnancy, and these EVs may serve as potential markers for various fetal and maternal pathologies during pregnancy [104–106]. EVs, like those obtained from umbilical cord blood, neonatal blood, or even urine, serve as markers for neonatal pathologies, particularly in prematurity and during the perinatal adaptation period, from birth until approximately 4 weeks after delivery [107].

In our case, as we did not have access to and analyze umbilical cord blood [108], we must assume that the EVs forming immunocomplexes found in the blood of newborns do not originate from the exchange of mother-fetus EVs through the placenta. This assumption is based on the short half-life of EVs in the circulatory system, as estimated in experimental studies [108]. The first sample analyzed from the blood of the infants was taken one month after birth during the first CD screening, as these infants were born to mothers with a history of infection. Therefore, the EVs detected in the infants’ serum, identifiable by antibodies from anti-*T*. *cruzi* immune serum, either originate directly from the infected children due to transplacental infection or may have maternal origin through breastfeeding.

The presence of EVs in colostrum [109] and breast milk has been associated with infant development [110]. Exosomes derived from breast milk have functions related to the maturation of the immune system [111], contributing to the increase in the number of regulatory T cells in peripheral blood, possibly to regulate immune tolerance [112]. It has also been demonstrated that exosomes derived from breast milk promote the proliferation of intestinal epithelial cells [113].

On the other hand, maternal immunoglobulins, primarily IgGs and IgA, present in colostrum and breast milk [114], contribute to maintaining immunity during infancy, as well as tolerance to intestinal bacterial flora and even the transfer of vaccine-induced antibodies to protect both the mother and the child from infectious diseases [115,116]. The passage of intact IgGs into the newborn’s circulatory system, after being ingested with breast milk, occurs through the intestine with the involvement of FcRN receptors to which IgGs bind [117,118]. The receptor binds immunoglobulin G (IgG) and albumin, retrieving them from degradation and transporting these ligands through polarized cellular barriers via a pH-dependent binding and release mechanism. These processes ensure the distribution and high levels of IgG and albumin throughout the body. These receptors are present in mucous membranes and particularly in the polarized cells of the intestinal wall [117,119], which facilitate the passage of IgGs from the intestinal mucosa to the newborn through transcytosis [120,121]. Monomeric IgGs and IgG immunocomplexes can be transported from either the apical or basolateral side of mucosal cells, where these receptors are located, to acidic pH endosomes, and the subsequent release of the receptor on the opposite cell surface in response to extracellular neutral pH. Different IgG immunocomplexes can pass through mucosal barriers via transcytosis [122], facilitating the mechanism of transcytosis for the passage of certain microorganisms forming immunocomplexes through mucous membranes and facilitating infection [123]. It is through this transcytosis mechanism that EVs containing *T*. *cruzi* antigens and forming immunocomplexes with IgGs could pass into the newborn’s bloodstream when breastfeeding by mothers with a history of infection.

In the present study, the active presence of the parasite was detected in mothers through the recognition of circulating EVs containing parasite antigens by anti-*T*. *cruzi* serum. However, in newborns, a decrease in absorbance was observed at 9 months post-birth, including in case 16, where the decrease was more pronounced although both the mother and the baby had been diagnosed as positive by PCR at one-month post-birth and were undergoing treatment with benznidazole. Also, breastfeeding had ceased at two months post-birth, which could have contributed to the decline in recognition of circulating parasite antigens, in the form of immunocomplexes with parasitic material. In case 10, the increase in absorbances observed at 9 months post-birth could be attributed to a real *T*. *cruzi* infection not detected by the diagnostic methods used and later confirmed. In the remaining infants, the decrease in recognition of *T*. *cruzi* circulating exovesicle antigens may correlate with the diminished or lack in breast milk intake over time after birth.

## Conclusion

In conclusion, the ease, minimal equipment requirement, and low cost of isolating EVs forming immunocomplexes, primarily using serum protein concentrators, and *T*. *cruzi* antigen detection in EVs should be applied in cases where evidence of active parasite forms is required, both in patients in the chronic phase and in cases undergoing treatment. To ensure detection in newborns, it would be necessary to apply it to umbilical cord blood at birth or take precautions, i.e., to stop breast milk intake a few days before conducting the test to ensure the parasitic origin of circulating immunocomplexes in the serum of these newborns.

## Supporting information

S1 Fig EVs quality and quantity control. A. NTA results of the total EVs obtained from the sera by ultracentrifugation. B. NTA results of the sera EVs obtained by protein concentrators. C. Transmission electron microscopy of the sera EVs purified by filtration/ultracentrifugation. The arrows show the EVs. The measuring bar 500 nm. D. Transmission electron microscopy of the sera EVs purified by the protein concentrators. The arrows show the EVs. The measuring bar 200 nm.
(DOCX)

S2 Fig Graphic representation showing the protein load of sera EVs samples obtained by protein concentrators and vesicles purified by ultracentrifugation. The red is EVs obtained by filtration with protein concentrators, while the blue represents the proteins obtained by ultracentrifugation. The blue line is the mean proteins of the samples obtained of the filtration procedure. The red line the mean of the samples obtained by filtration procedure. Each serum sample is represented on the x axis.
(DOCX)

S3 Fig A. Electrophoresis in SDS PAGE of T. cruzi EVs and Immunocomplexes with EVs obtained from a pool of sera from cardiac patients. B. Antigenic recognition against MASP-SP by immunosera obtained against the synthetic peptide in the immunocomplexes obtained from patient sera. C. Antigen used in immunization against MASP-SP peptide. Four copies of the synthetic peptide were bound by branched Lysines.
(PDF)

S4 Fig WB analysis of antigenic recognition by sera from patients in Panama. A. Results of patients from the urban area. B. Results of patients from the rural community studied. The WB analysis reveals distinct antigenic bands in positive sera (25, 30, 45, 52, 70 kDa).
(TIF)

S1 Table Data on the adults studied, age range, sex, origin, and diagnostic tests used and their results. (XLSX)

S2 Table Data on positive mothers and newborns. (XLSX)

S3 Table Data of the negative Controls used. (XLSX)
