# Supplementary material for: Circulating extracellular vesicles in sera of chronic patients as a method for determining active parasitism in Chagas disease
Source: PLoS Negl Trop Dis. 2024 Nov 20;18(11):e0012356. doi: 10.1371/journal.pntd.0012356 (PMC11616892; doi:10.1371/journal.pntd.0012356)
Supplement: S1 Fig — A. NTA results of the total EVs obtained from the sera by ultracentrifugation. B. NTA results of the sera EVs obtained by protein concentrators. C. Transmission electron microscopy of the sera EVs purified by filtration/ultracentrifugation. The arrows show the EVs. The measuring bar 500 nm. D. Transmission electron microscopy of the sera EVs purified by the protein concentrators. The arrows show the EVs. The measuring bar 200 nm. (DOCX) [file pntd.0012356.s001.docx]

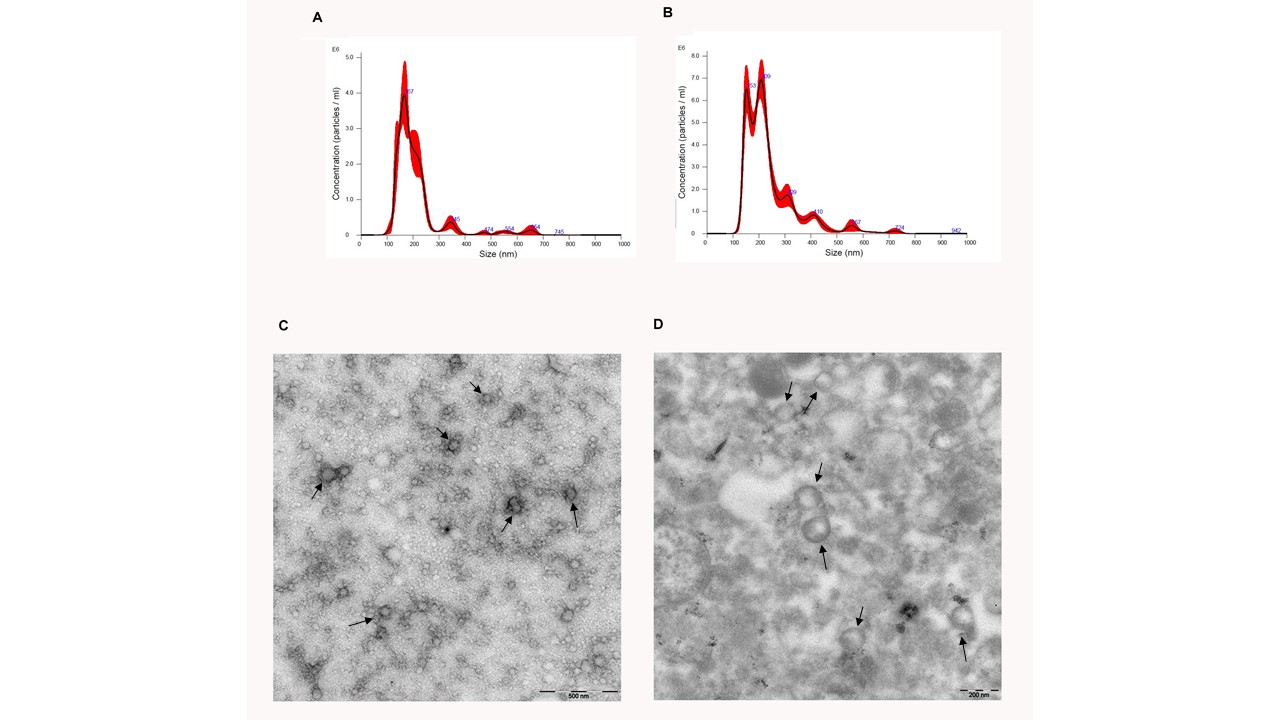


Figure S1.

A.- NTA results, of the total EVs obtained from the sera by ultracentrifugation

B.- NTA results of the sera Exovesicles obtained by protein concentrators

C.- Transmission electron microscopy of the sera Exovesicles purified by filtration/ultracentrifugation. The arrows show the exovesicles. . The measuring bar 500nm.

D.- Transmission electron microscopy of the sera exovesicles purified by the protein concentrators. The arrows show the exovesicles. The measuring bar 200 nm.
