# Supplementary material for: Circulating extracellular vesicles in sera of chronic patients as a method for determining active parasitism in Chagas disease
Source: PLoS Negl Trop Dis. 2024 Nov 20;18(11):e0012356. doi: 10.1371/journal.pntd.0012356 (PMC11616892; doi:10.1371/journal.pntd.0012356)
Supplement: S2 Fig — The red is EVs obtained by filtration with protein concentrators, while the blue represents the proteins obtained by ultracentrifugation. The blue line is the mean proteins of the samples obtained of the filtration procedure. The red line the mean of the samples obtained by filtration procedure. Each serum sample is represented on the x axis. (DOCX) [file pntd.0012356.s002.docx]

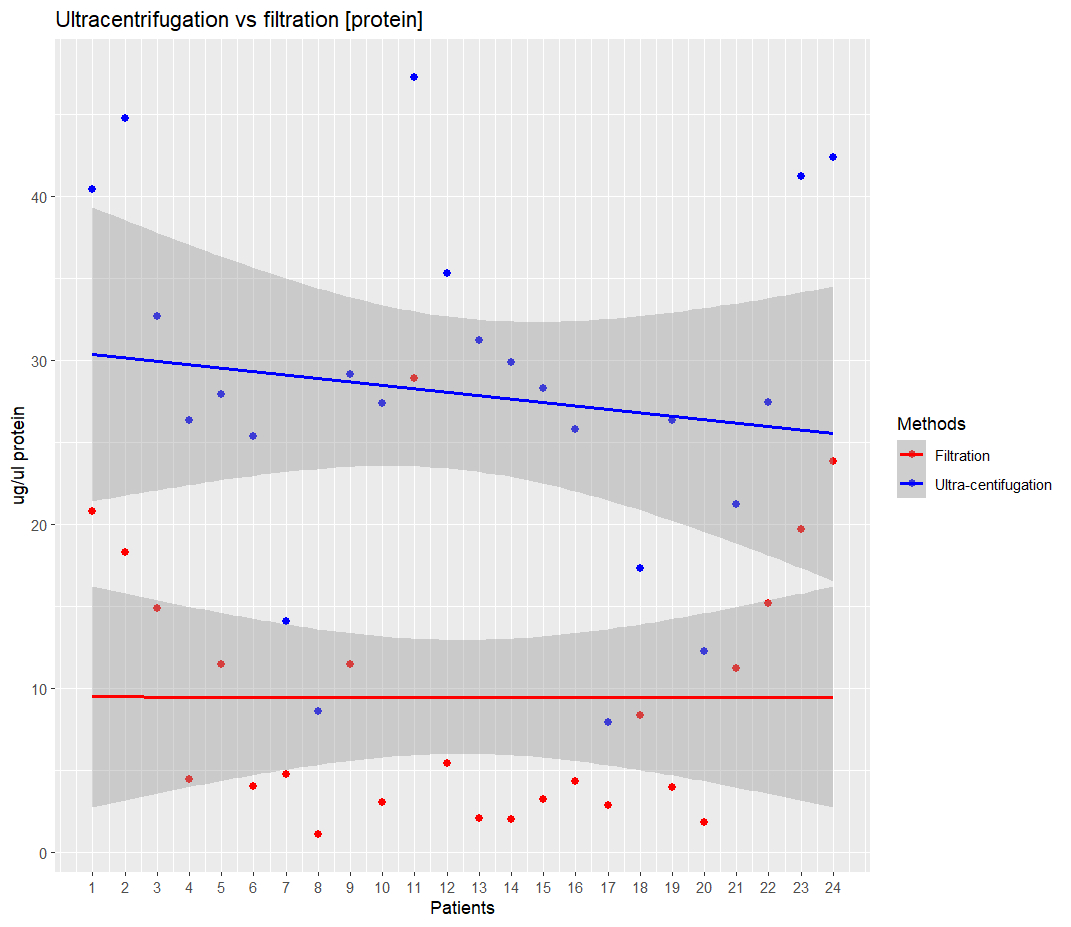


Figure S2.-

Graphic representation showing the protein load of sera EVs samples obtained by protein concentrators and vesicles purified by ultracentrifugation. The red is exovesicles obtained by filtration with protein concentrators, while the blue represents the proteins obtained by ultracentrifugation. The blue line is the mean proteins of the samples obtained of the filtration procedure. The red line the mean of the samples obtained by filtration procedure. Each serum sample is represented on the x axis.
