# Supplementary material for: Circulating extracellular vesicles in sera of chronic patients as a method for determining active parasitism in Chagas disease
Source: PLoS Negl Trop Dis. 2024 Nov 20;18(11):e0012356. doi: 10.1371/journal.pntd.0012356 (PMC11616892; doi:10.1371/journal.pntd.0012356)
Supplement: S3 Fig — A. Electrophoresis in SDS PAGE of T. cruzi EVs and Immunocomplexes with EVs obtained from a pool of sera from cardiac patients. B. Antigenic recognition against MASP-SP by immunosera obtained against the synthetic peptide in the immunocomplexes obtained from patient sera. C. Antigen used in immunization against MASP-SP peptide. Four copies of the synthetic peptide were bound by branched Lysines. (PDF) [file pntd.0012356.s003.pdf]

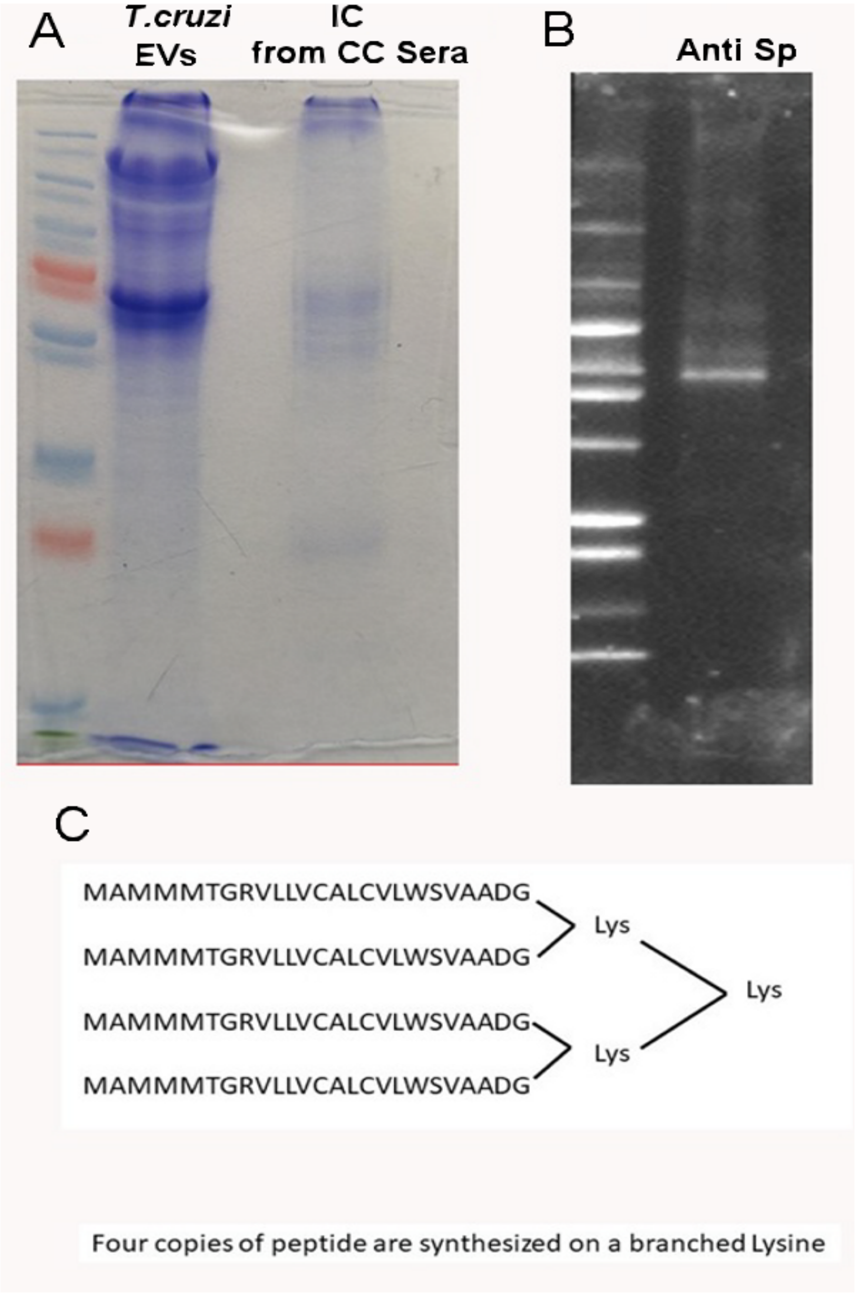

Figure S3.

A. - Electrophoresis in SDS PAGE of T cruzi EVs and Immunocomplexes with EVs obtained from a pool of sera from cardiac patients.

B.- Antigenic recognition against MASP-SP by immunosera obtained against the synthetic peptide in the immunocomplexes obtained from patient sera.

C.- Antigen used in immunization against SP peptide.

Four copies of the synthetic peptide were bound by branched Lysines.
