# Supplementary figures and images for: Circulating extracellular vesicles in sera of chronic patients as a method for determining active parasitism in Chagas disease
Source: PLoS Negl Trop Dis. 2024 Nov 20;18(11):e0012356. doi: 10.1371/journal.pntd.0012356 (PMC11616892; doi:10.1371/journal.pntd.0012356)

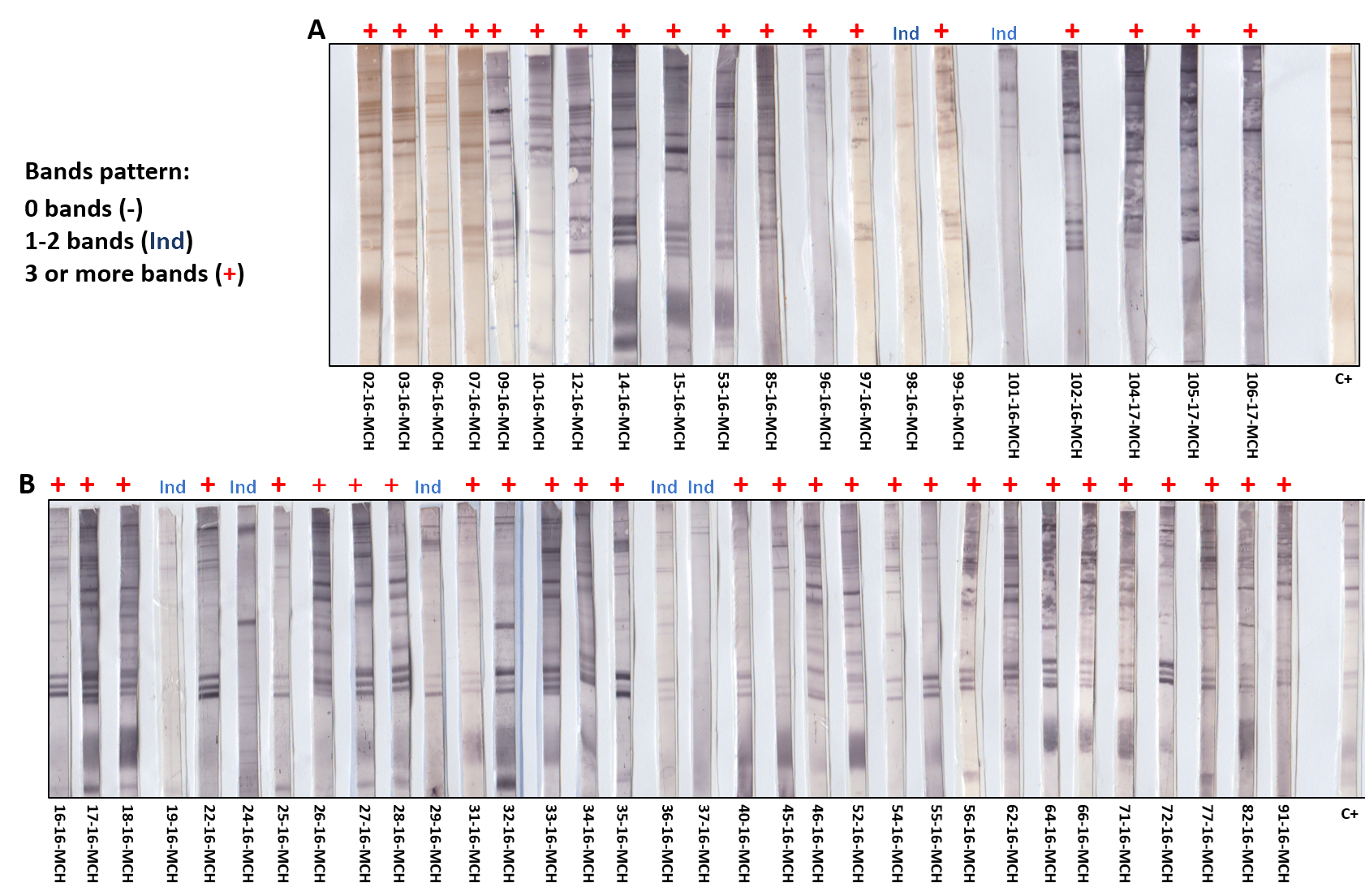

Supplement: S4 Fig — A. Results of patients from the urban area. B. Results of patients from the rural community studied. The WB analysis reveals distinct antigenic bands in positive sera (25, 30, 45, 52, 70 kDa). (TIF) [file pntd.0012356.s004.tif]
